# The Impact of Retirement on Happiness and Loneliness in Poland—Evidence from Panel Data

**DOI:** 10.3390/ijerph18189875

**Published:** 2021-09-19

**Authors:** Anita Abramowska-Kmon, Wojciech Łątkowski

**Affiliations:** Institute of Statistics and Demography, SGH Warsaw School of Economics, 02-554 Warsaw, Poland; wlatko@sgh.waw.pl

**Keywords:** retirement, happiness, subjective quality of life, loneliness, panel data, Poland

## Abstract

This paper examines the impact of retirement on people’s subjective quality of life, as expressed by their levels of happiness and loneliness, in Poland. We analysed five waves of the Social Diagnosis panel survey conducted between 2007 and 2015. To account for unobserved individual heterogeneity, we employed fixed effects ordered logit models and fixed effect logistic models for the panel data. We found that the respondents’ happiness levels did not change after they retired, and that the introduction of interactions between retirement and employment did not alter these findings. However, the results of the loneliness model showed that the probability of being lonely increased among males after retirement. Second, the outcomes of interactions between retirement and employment suggested that not working after retirement increased the likelihood of being lonely among men, whereas engaging in bridge employment decreased the chances of being lonely among men. These findings may indicate that combining retirement with employment may be a source of social interaction, which can provide protection against loneliness, and which may, in turn, be positively related to other factors (i.e., subjective quality of life, health status, and mortality).

## 1. Introduction

Retirement is one of the major life events that affects people’s subjective quality of life/subjective well-being (SWB). Although intensive research on this topic has been conducted, the evidence on the impact of retirement on life satisfaction has been mixed [1]. Retirement has been analysed from different angles, with many scholars investigating the effect of retirement on an individual’s subjective quality of life. As van Solinge and Henkens noted, researchers usually adopt an individualistic approach to studying retirement, even though it can be perceived not only as an occupational career transition but as a family transition that is also experienced by couples [2]. Research on retirement can, for example, investigate whether it is voluntary (e.g., [3]), whether a retiree takes up bridge employment [4], and whether an individual retired at the regular statutory age or early [5]. An individual may transition to retirement while unemployed or while employed [6]. Similarly, the impact of retirement on individuals can by scrutinised from the short-term or the long-term perspective [7,8]. Furthermore, job (dis)satisfaction appears to play a role in the transition to retirement. It has, for example, been shown that having low-quality work is associated with a higher probability of becoming unemployed, and of retiring early through either full or partial retirement [9]. Moreover, as mortality has decreased in recent decades, and life expectancy has reached unprecedented levels, the length of time people spend in retirement has been increasing as well, with a growing number of individuals spending 20, 30, and even 40 years in this stage of life. Thus, the duration of this life phase may have an impact on older people’s subjective quality of life, and on the broader society and social policy.

All of these situations and factors, as well as individual socio-demographic and economic characteristics, personality traits, and experiences, may mitigate or enhance the relationship between retirement and subjective well-being. For example, having a higher level of education may enable an individual to secure a better job and more financial resources, which can, in turn, lead to greater life satisfaction [10,11,12]. Similarly, married adults are happier than those living without a spouse [10,13,14], while people who have a disability or long-term health problems tend to be less satisfied with life [10,15,16]. In addition, the assets people accumulate over their life course (i.e., financial resources, health (dis)advantages) may influence their process of adapting to retirement, and, thus, their subjective quality of life [17,18,19]. The results of analyses of the relationship between personal traits and subjective well-being in the context of the transition to retirement have shown that certain personality characteristics (i.e., openness) may be associated with increased SWB because they ease the process of adaptation to a new stage of life [20].

In the temporal process model of retirement, the transition from work to retirement is conceptualised as a process consisting of three sequential phases: retirement planning, retirement decision-making, and retirement transition and adjustment [21]. In this study, we focused on the last phase in order to gain a better understanding of how transitioning to retirement affects an older person’s subjective quality of life, as approximated by the happiness level and loneliness. Thus, the main objective of this study is to analyse the impact of retirement on happiness and loneliness levels in Poland. In particular, we seek to investigate whether having a job after retirement influence SWB and loneliness. For this purpose, we draw on data from five waves of the Social Diagnosis: the conditions and quality of life of Poles panel survey. We employed fixed effects logit models and fixed effect ordered logistic models with the blow-up and cluster (BUC) estimator for panel data. Unlike cross-sectional data, panel data allow us to control for unobservable heterogeneity by gathering analogous information on individuals at different points in time. Thus, the changes in subjective quality of life may be interpreted by examining the changes in respondents’ characteristics or situations.

To the best of our knowledge, this topic has rarely been investigated for Poland, especially with the use of panel/longitudinal data [22]. Moreover, Poland, together with other Central-Eastern European states, belongs to a group of countries with rather low subjective quality of life levels [23,24,25,26]. Thus, additional research on the determinants and the effects of different life decisions, events, and situations on subjective quality of life in Poland is needed. Furthermore, this analysis can help to broaden our understanding of the retirement process as one of the key changes in the individual life course, and of the impact of retirement on individual psychological well-being, as such insights are crucial to efforts to prolong working life in order to reduce the negative consequences of the population ageing process in Poland.

In the next section, we provide a review of the literature on the impact of the transition to retirement on subjective quality of life among older workers and among older people in the early stages of retirement. Then, we describe the data and methods we used in the study. Finally, we present our empirical results, and our conclusions. In this paper, in line with other studies on this topic [27], we use interchangeably the terms/concepts of subjective quality of life, psychological well-being, life satisfaction, and happiness, as they are strongly interrelated, and characterise different aspects of quality of life.

## 2. Retirement and Subjective Quality of Life: A Literature Overview

### 2.1. Transition to Retirement and Subjective Quality of Life—A Review of Theories

The existing literature has offered a number of theoretical perspectives on how retirement affects an individual’s well-being. The role theory ([28] after [29]) views retirement as a transition from work roles (e.g., the worker role, the organisational member role, the career role) to non-work roles (i.e., the family and the community member roles). The effect of moving from employment to retirement may be either negative or positive, depending on an individual’s level of involvement in work roles relative to level of engagement in other roles. People who invested a lot in their work-related roles, or for whom work was an important part of their identity, may experience lower levels of well-being, anxiety, or depression when they retire. By contrast, people who found their job stressful and burdensome, or who wanted to be more involved in their family member or community member roles, may experience retirement as a positive change, or even as a relief [29].

According to the continuity theory [30], individual patterns of behaviours, activity profiles, and social engagement are consistent over time, which suggests that people tend to follow familiar strategies at different moments of life; hence, continuity is the main adjustment strategy [31]. This means that the process of retirement consists not only of the termination of paid work, but also of many different individual characteristics (i.e., self-esteem) and of the actions a person takes to adjust to new circumstances, which can, in turn, enable him/her to preserve the level of subjective quality of life after retirement [32]. Therefore, retirement is not necessarily a negative event, especially if an individual is able to maintain the lifestyle patterns established before leaving employment, or if the individual perceived retirement as part of a prior plan for later life.

Another theoretical framework for analysing retirement is the life course perspective, which focuses on the context in which people live their lives [33,34]. This perspective regards retirement as both a transition (a change in an individual’s status from employed to retired) and a trajectory (the development of an individual’s life in the postretirement period) that is embedded in contextual circumstances (the individual’s attributes, current and past status, and one’s roles and social context). Consequently, in accordance with the linked lives paradigm, an individual’s perception of retirement experience (whether positive or negative) may vary by, for example, whether a person is married or how strongly he/she identifies with family roles [29].

Finally, the adjustment to retirement can be viewed as a resource-based dynamic process [1,29]. The point of departure for this theory is the observation that retirees do not follow a uniform adjustment pattern during the retirement process [29]. Within this framework, changes in the trajectory of an individual’s life satisfaction after retirement are a reflection of the gains and losses in personal resources that accompany the transition to retirement. These resources can be physical, cognitive, motivational, financial, social, or emotional, and can be accumulated over time. Moreover, these resources are influenced by a variety of antecedents, including variables at the macro level, the organisational level, the job level, the household level, and the individual level [1].

### 2.2. Retirement and Life Satisfaction: Previous Findings

Retirement is considered one of major late midlife events that affect subjective well-being. However, the results of previous research on the impact of retirement on SWB have been inconsistent [1,35]. Some studies have found that retirees have higher levels of life satisfaction than workers, especially in the early stages of retirement [6,7,36,37,38]. It has been argued that retirees often enjoy having free time and feel released from the pressures of employment [36]. Other researchers have found that retirement has no effect on subjective well-being [20,22,32,39,40], or on the probability of becoming depressed [5]. The sense of control over the transition appears to be of great importance. The association of retirement with loneliness depends on whether the transition to retirement was voluntary or involuntary, with involuntary retirement being associated with higher levels of loneliness [41]. There is also evidence that involuntary retirement has adverse effects on a person’s life satisfaction [4,42].

The transition to retirement cannot be seen as a uniform process, because individual life histories can vary greatly. The assumption that the transition to retirement can take different forms was confirmed in a study based on data from the US Health and Retirement Survey [29]. The results showed that of the retirees in the study sample, around 70% experienced no change in psychological well-being, less than 5% experienced a positive change in well-being, and 25% suffered a decline in well-being. Similar percentages were found by Pinquart and Schindler [43] using German data: of the retirees in their sample, 15% experienced an increase in life satisfaction, 9% experienced a decrease in life satisfaction, while the majority reported no significant change in well-being. Moreover, Sohier et al. demonstrated that while life satisfaction does not change immediately after retirement, it is diminished two years after this transition [40]. Similar results were obtained by Heller-Sahlgren, who found that while retirement has no effect on mental health over the short term, the depression levels of retirees increase significantly over the long term [44]. Similarly, Segel-Karpas, Ayalon, and Lachman observed that depressive symptoms increase after retirement [45].

Against this background, scholars have long been interested in identifying the factors that influence the differences in life satisfaction levels during the retirement transition. In their review, Wang et al. summarised the variables that influence how well people adjust to retirement using five categories: individual attributes, preretirement job-related variables, family-related variables, retirement transition-related variables, and postretirement activities [1]. Szinovacz argued that financial status, health, and individual attributes are the main attributes associated with well-being in retirement [46]. Both of these studies have received considerable attention in the literature.

One of the most important consequences of transitioning to retirement is a considerable decrease in economic resources [47], which may have a negative impact on subjective well-being, as a favourable financial situation has been shown to contribute to higher levels of life satisfaction after retirement, mostly among men [48]. However, this effect may be mitigated or reinforced by the wealth a retiree accumulated during one’s working life. In general, the better the financial status of an individual is, the lower the risk that a person will experience a decline in life satisfaction during the transition to retirement [7,43]. Similarly, Yeung observed that retirees who can maintain the level of resources they had while working do not experience a decrease in well-being after retirement [47]. In contrast, Calvo, Haverstick, and Sass found that wealth has no significant effect on the happiness of retirees [49].

Transitioning to retirement means that an individual experiences a change in daily activities, and is able to choose between leisure activities, volunteer work, and paid work [21]. It has been shown that retirement tends to increase the proportion of time people spend on leisure, social, and cultural activities [50], as well as on domestic tasks and volunteer work, which can include caring for family members or being involved in organisations [51]. Bonsang and Klein argued that retirement has two effects: a sizable positive effect on satisfaction with free time, and a sizable negative effect on household income. They therefore concluded that the average effect of retirement on life satisfaction is negligible [42].

The association between an individual’s health status and retirement is bidirectional. On the one hand, physical and mental health are strong predictors of the decision to retire [45,52,53] and of retirement timing; i.e., individuals who are in better physical health are more likely to retire later [54,55] and to engage in postretirement paid work [21]. Moreover, an opportunity to retire early may have positive effects on a person’s mortality and health [56]. On the other hand, the effect of retirement on an individual’s health status depends on personal characteristics and life history. In the retirement adjustment process, physical health is considered an important resource that accumulates over the life course. Therefore, individuals with worse health are at risk of experiencing a decline in life satisfaction when transitioning to retirement [17,18,43,48]. In general, research on the linkages between health and (early) retirement has found that retirement does not increase the risk of health deterioration. Indeed, it has been shown that retirement contributes to better self-reported health and reductions in activity limitations for both men and women [56,57,58,59]. However, these patterns appear to differ across socio-economic groups. For example, some studies observed improvements in health status for individuals of all educational levels [59], while other studies found improvements only for individuals with high socio-economic status [60]. Similar results were reported by Gorry et al., who found that self-reported health increases after retirement, especially over the longer term [39]. A potential explanation for this finding is that retirees make positive changes in their healthy behaviours (more physical activity, less smoking, a better diet, etc.) and are more engaged in social activities. As being in better health enhances subjective well-being [15,16], if a person’s health improves after retirement, life satisfaction may increase as well. Similarly, Calvo et al. observed that improvements in health are associated with higher levels of happiness and enjoyment of life, and with lower levels of loneliness, depression, and sadness [49].

The relationship between preretirement and postretirement employment and subjective quality of life is also worth examining. While it is common for retirees to continue to engage in paid work and to acquire pension benefits after the transition to retirement, the relationship between working and SWB in retirement remains unclear. For example, it has been shown that individuals who continue to work after retirement are as satisfied with life as those who stopped working completely [40]. Other studies have found the opposite effect; i.e., that bridge employment enhances the life satisfaction levels among people who retired voluntarily and reduces the decline in life satisfaction among people who retired involuntarily [4]. It is worth noting that this relationship depends on the cultural context and the employee’s qualifications [61]. There is also evidence that having a paid job after retirement lowers depression levels [32], although this effect appears to be moderated by whether the work is voluntary or involuntary. It has, for example, been shown that pensioners who are forced to prolong their working life after retirement because of their circumstances (i.e., financial issues) are less satisfied with life than those who do not continue to work [62]. Moreover, often even if a retiree is working voluntarily, the job may be of low quality or beneath one’s qualifications, which may reduce the individual’s subjective well-being after retirement. In terms of preretirement job-related variables, whether an individual was unemployed before transitioning to retirement, and the challenges and stress levels faced in the workplace, can affect the emotional consequences of retirement. It has been observed that retiring from unemployment may be more beneficial than from retiring from employment, because it can improve the individual’s income and status [6,43]. In addition, it has been shown that retirees who retire from highly physically demanding jobs are more likely to experience positive changes in psychological well-being than retirees who retire from less physically demanding jobs [29,63]. There is also evidence that job dissatisfaction contributes to greater satisfaction with life after retirement [48]. In this context, the findings of Damman et al. are interesting, as they pointed out that retirees can miss work for different reasons depending on their career path in midlife [64]. They found, for example, that retirees who had a steep upward career path in midlife were less likely than those who did not experience upward mobility to miss their money/income, but were more likely to miss their status.

Social networks are also crucial elements of the decision to retire, the timing of retirement, and the impact of retirement on well-being. The size of a person’s social network and the intensity of contacts with its members are positively related to early retirement [65]. Moreover, the composition of an individual’s social network, particularly the presence of a spouse as the person with whom the person has the most frequent contact, increases the risk of early retirement. In addition to having a spouse, the timing of retirement is influenced by a person’s employment status [65]. Having a working spouse lessens the chances of retiring earlier, while having a spouse who does not work has the opposite effect. It has been shown that for older women in particular, their social contacts in the period prior to retirement are especially crucial for their SWB [48]. Among the family-related variables, the quality of the marital relationship stands out as a predictor of satisfaction with retirement [2]. An individual’s marital history appears to play a role as well, as a divorced retiree without a partner is especially likely to have difficulties adjusting to the social changes associated with the loss of his/her work role [64].

### 2.3. Retirement in Poland

Poland, which is the focus of our analysis, can be described as a country with an early retirement tradition. Two major pension reforms aimed at increasing the actual and the statutory retirement age have been introduced in Poland. The pension reform of 2009 reduced the options for retiring early by eliminating a number of early retirement schemes. Additionally, actions were taken to reduce eligibility for disability benefits [66]. As a result, the employment rate in Poland for people aged 55–64 increased from 31.6% in 2008, to 44.3% in 2015, and to 49.5% in 2019. However, the employment rate for older people in Poland still lags behind the EU-28 and the OECD average. The reform of 2013 was expected to improve the adequacy of pensions and the financial stability of the pension system. It introduced unification of the statutory retirement age for men and women with a formula designed to gradually increase it to 67 years. Before 2013, the statutory retirement age was 60 years for women and 65 for men. From 2013 onwards, the pension eligibility age was increased gradually by four months per year, and is on track to reach 67 years in 2020 for men and in 2040 for women [67]. In 2017, changes in the statutory retirement age were introduced that lowered it to 60 years for women and to 65 years for men. Although the average age at which people started receiving old-age pension benefits increased from 61.5 years for men and 58 years for women in 2005 to 62.8 years for men and 60.6 years for women in 2018 [68], this level is still below the statutory age for men. From a comparative international perspective, the effective labour force exit age in Poland is lower than the European Union and the OECD average, especially for women. Additionally, the Polish population is ageing fast, as the share of people aged 65 or older in the total population is projected to increase from 17.7% in 2019 to 33.9% in 2060 [69].

While Poles retire early for many different reasons, the desire to retire as early as possible is widespread in the country [70,71]. As a consequence, the employment rates of people aged 50–64 are very low in Poland, and unemployment in this group often leads to complete inactivity rather than to reemployment [72]. Thus, a person’s motives for retirement and individual characteristics and history may influence the decision about when to retire, and the effects of retirement on subjective quality of life.

Based on the literature review described above, we propose the following research hypotheses:

(1) Retirement transition does not affect the happiness level and loneliness among retiring Poles.
(2) Continuing professional work after retirement is beneficial for subjective well-being/reduces loneliness.
(3) The effect of retirement on subjective quality of life/loneliness is similar for men and women in Poland.

## 3. Materials and Methods

### 3.1. Data

For our analysis, we used data from the Social Diagnosis: living conditions and quality of life of Poles panel survey, which was carried out in Poland in 2000–2015 [73]. This was a comprehensive survey that covered various aspects of the living conditions of households and their individual members. Specifically, it collected data on the economic aspects (labour market status, income, material situation, etc.) as well as the non-economic aspects (psychological well-being, happiness, satisfaction with different domains of life, lifestyle, health care, education, unhealthy behaviours, participation in culture and social life, use of modern technologies of communication, etc.) of the lives of individuals. A total of eight waves of this survey were carried out (in 2000, 2003, 2005, 2007, 2009, 2011, 2013, and 2015). In all eight waves conducted in the 2000–2015 period, data on more than 62,500 respondents aged 16 years or older were collected [73]. However, for the purposes of our analysis, we limited the sample to the respondents aged 55–69 who participated in the last five waves of the survey (i.e., in 2007–2015), and who transitioned to retirement over this period. In our analysis, we omitted the data from the first three waves of the Social Diagnosis Survey (editions 2000, 2003, and 2005), because they did not allow us to determine the retirement status of the respondents unequivocally. We also removed the observations with missing values for all of the variables in the models. Importantly, our sample was restricted to respondents who participated in at least two waves of the study, which was conditioned by the employed models. The numbers of the respondents in the subsequent waves changed due to the attrition effect and the expansion of the sample, which resulted in an unbalanced panel. The final sample contained information on 1503 observations (567 male and 936 female observations) across all five waves. Table A1 (in Appendix A) presents the number of observations in the final sample in all five waves.

### 3.2. Variables in the Models

#### 3.2.1. Dependent Variable

In our analyses, we concentrated on happiness as a dimension of subjective quality of life, which was based on the following question “All in all, how would you assess your life in recent times—would you say you are…”, with four possible answers: 1—very happy, 2—rather happy, 3—rather unhappy, and 4—very unhappy. However, for the purposes of our analyses, we changed the order so that 1 signified very unhappy and 4 signified very happy. This variable captured temporal changes in happiness. The loneliness variable was based on the question “Do you feel lonely even though you do not want to?”, with two possible answers: yes (1) and no (0).

#### 3.2.2. Control and Explanatory Variables

In order to answer our research questions, we included a set of explanatory variables in the models that encompassed not only the basic socio-demographic and economic characteristics of the individual respondents, but also the variable describing their retirement status. In particular, we incorporated the following variables into the basic models: sex (ref. men), age (continuous), place of residence (ref. urban area), presence of disability (ref. without disability), partnership status (ref. without a spouse/partner), living arrangements (ref. living alone), educational level (ref. primary and lower), employment status (ref. not in employment), satisfaction with the financial situation of one’s family (continuous), and satisfaction with one’s health status (continuous). Our key explanatory variable describing the retirement status of the respondents was derived from the information on the reason for economic inactivity; i.e., based on their statements that they were not working because they were retired. However, a share of the retired respondents could work, as the employment status of these respondents showed. The reference category for the retirement status was “not retired”.

The categorical variables after necessary transformations were included as a set of binary variables with the reference categories that are described above. Three variables (age, satisfaction with the financial situation of one’s family, and satisfaction with one’s health) were treated as continuous. Table A1 (in Appendix A) presents the descriptive statistics for all the covariates in the models in all five waves.

### 3.3. Methods

In our approach, we used panel data to determine the effects of different variables on happiness and loneliness, which allowed us to control for unobserved heterogeneity among individuals (for example, their unobservable inclination for pessimism or optimism). Life satisfaction/happiness may be introduced into econometric models as a latent variable measured on a continuous scale (Y*), whereas what we observed is an answer to the survey question (Y) measured on an ordinal scale. Thus, in this estimation procedure, the ordinal character of the dependent variable (Y) with values from 1 (very unhappy/dissatisfied) to 4 (very happy/very satisfied) was taken into account.

In this paper, we estimated a fixed-effects ordered logit model, which may be expressed as follows:

(1) $$y_{it}^{*}=x_{it}\beta +\alpha_{i}+\epsilon_{it},\;i=1,\ldots ,\;n,\;t=1,\ldots ,\;T$$

where y*_it_ stands for unobserved happiness level, *x_it_* stands for a vector of observed individual characteristics, *β* stands for a vector of coefficients, $\alpha_{i}$ stands for individual specific intercepts, and $\epsilon_{it}$ stands for a time-varying unobservable term that is independent and identically distributed with a standard logistic cumulative density function.

The observed happiness/life satisfaction (*y_it_*) is related to the modelled unobserved y*_it_ in the following way:

(2) $$y_{it}=\{\begin{array}{c} 1\;\mathrm{if}\;y_{it}^{*}\leq \mu_{1}\\ 2\;\mathrm{if}\;\mu_{1}<y_{it}^{*}\leq \mu_{2}\\ 3\;\mathrm{if}\;\mu_{2}<y_{it}^{*}\leq \mu_{3}\\ 4\;\mathrm{if}\;\mu_{3}<y_{it}^{*} \end{array}$$

Fixed effects models are often used in social sciences, including in analyses of the effects of retirement [48], because they can be used to estimate casual effects by controlling for unobserved individual heterogeneity. While several estimators are used in model estimation, in this study, we employed the blow-up and cluster (BUC) estimator proposed by Baetschmann et al. [74,75]. It has been proven that the BUC estimator has good properties, and is as efficient as more complex estimators. This estimator was implemented in a STATA command *feologit*, which allows for estimation of other model elements (such as marginal effects, odds ratios, etc.). In this method of estimation, is it implied that each individual has different thresholds; thus, the estimates generated by this method are not provided in the tables with the results. Moreover, the program excludes respondents who were observed only once or did not differ with respect to a dependent variable between waves. In contrast, for a binary dependent variable describing loneliness, we employed a fixed-effects ordered logit model.

For all the dependent variables, we estimated the models for the total population aged 55–69, and for males and females separately (Model A) in order to investigate the differences between the sexes. Moreover, to investigate the relationship between retirement status and employment status, we estimated similar models, but with the interaction between the two covariates (Model B).

## 4. Results

The estimation results of the models for happiness are presented in Table 1. In Model A, we introduced separate variables describing retirement status and employment status, while in Model B, we incorporated the interactions between those variables in order to account for the differences between individuals who continued working after retirement. Most of the coefficients were found to be significant at the 0.01 level (and, in some cases, at the 0.1 level). Generally speaking, the effects of the majority of the control variables in all of the models were consistent with the findings described in the literature devoted to subjective well-being; however, some differences between the models were detected.

As for the relationship between retirement and the happiness level, the results of Model 1A showed that among the people who retired in the analysed period, their level of happiness did not change significantly after they retired (Table 1). Similar outcomes were obtained in Model 1B with the interaction terms between retirement status and employment status: neither stopping work nor continuing to work after retirement affected the respondents’ levels of happiness after they retired. These findings may suggest that there are different groups of people who experience the transition to retirement in different ways, as shown by, for example, the authors in [43]. Thus, the mean effect of retirement on SWB for a studied population may be insignificant or minor.

It is also worth mentioning the results we obtained for the other variables in the models. For instance, we found that partnered men and women were happier than those living without a partner, which is in line with the results of previous research [10,76,77,78]. Having a better financial situation (assessed here subjectively) was found to be associated with higher levels of happiness in the analysed group, which is consistent with the findings of previous research [12,79]. Although the estimates for disability turned out to be insignificant, satisfaction with one’s health status was shown to contribute to higher levels of happiness, which confirms the outcomes of other analyses [15,80,81]. In addition, being employed was found to be positively related to happiness among females only.

A different picture emerged in Model 2, which included loneliness as a dependent variable (Table 2). The results of Model 2A showed that retirement increased the risk of loneliness among men, but not among women. A more detailed image can be observed when the interaction between retirement status and employment status is taken into account (Model 2B). Our outcomes demonstrated that males who retired and did not continue to work had a higher probability of being lonely than those who were not retired and were not working, while the results for females were insignificantly negative. Moreover, we found that the respondents who had retired and were still employed were less likely to be lonely than the respondents who were not working and not retired, and that this effect was bigger (and significant) for men. These findings suggest that after men retire, their social networks tend to shrink, which may lead to diminished social interactions, and, in turn, to higher levels of loneliness. To conclude, these results may indicate that there was a positive relationship between bridging employment and subjective quality of life among this group, as loneliness was related to depression, and thus to lower SWB.

## 5. Discussion

The aim of this paper was to analyse the relationship between retirement and happiness and loneliness among people aged 55–69 in Poland. To some extent, our results are in line with those reported in the literature review. In general, in the models without interactions (A), the happiness levels of the surveyed individuals did not change after they retired. A similar pattern was observed when the interactions between retirement and employment were introduced. We obtained more information from the models with loneliness as a dependent variable. First, retirement increased the probability of loneliness among males. Second, the interaction between retirement status and employment status showed that not working after retirement increased the likelihood of being lonely among men, while engaging in bridge employment decreased the chances of being lonely among men. This finding may suggest that combining retirement with employment may be a source of social interactions, which can protect people from loneliness, and which may, in turn, be positively related to other factors (i.e., subjective quality of life, health status, and mortality). In terms of our research hypotheses, our results allowed us to draw the following conclusions: retirement did not change happiness levels, but it did have an impact on loneliness, as men who continued working after retirement were less lonely, and individuals who were not working after retirement were more lonely than individuals who were not working and not retired.

This work contributes to the research on subjective well-being in retirement through its focus on Poland, a Central European country with a culture that promotes retiring as early as possible [70,71]. This is especially important in the context of the approach we used in our analysis of focusing on individuals for whom we could observe the transition to retirement. However, this analysis has some limitations. First, we used single-item questions as the dependent variables describing happiness and loneliness. Although they capture the studied phenomena quite well, more complex variables composed of different dimensions of subjective well-being or loneliness would be more appropriate and more informative in this context. Secondly, we excluded from the final dataset individuals who retired at younger ages (<55), and who may make up a substantial share of the original sample, as well as individuals who retired at older ages (>69). Moreover, we did not distinguish between early and regular retirement, which could help to differentiate the results and shed some light on the impact of early retirement on SWB among Poles. Therefore, future analyses should focus on this topic as well. Furthermore, since the study was carried out every two years, it was impossible to detect the exact time of the event of interest, which may have affected our results. In particular, it would be interesting to investigate the short-term and the long-term consequences of retirement on subjective well-being, and to thus verify the continuity theory [30].

Another issue is the endogeneity of retirement, which may be caused by many reasons. For example, health status may have impact both on retirement decision and subjective well-being, while the omitted variables, such as job satisfaction or caring responsibilities, may also influence simultaneously a withdrawal from the labour market and subjective quality of life. While the dataset we used in the analyses provided us with the main control variables, having additional information—such as information on preretirement job-related variables (e.g., job characteristics, job satisfaction), individual attributes (e.g., objective measures of health, family situation, caring obligations), motives for retirement, whether the retirement was or was not voluntary, and characteristics of bridge employment (i.e., a part-time or a full-time job)—could improve our understanding of the process of the transition to retirement in Poland. Unfortunately, the Social Diagnosis survey ended in 2015, but it could become an important source of information on subjective quality of life for Poles at all ages, including for those nearing the retirement age or in the early years of retirement. Furthermore, a longer panel would allow us to study the well-being trajectories of different subpopulations, and to evaluate whether any changes in happiness levels are temporary or stable over time. Moreover, such data could shed new light on the relationship between the transition to retirement and subjective quality of life in recent years among new cohorts of retirees, especially given the changes in the statutory retirement age and the growing social awareness of demographic changes, and of the consequences of increasing life expectancy on pension benefit amounts.

## 6. Conclusions

We believe that research on the transition to retirement is extremely important because this event triggers changes in many areas of a person’s life, including psychological well-being/life satisfaction and organisation of daily activities, as well as health status and mortality. These consequences may differ for different subpopulations, especially those who retired earlier or those with lower socio-economic status, which may require the development of different social policies aimed at improving the quality of life of disadvantaged populations. This is especially important in the context of efforts to prevent unequal ageing and to help individuals reduce their risk of detrimental outcomes, including poor health and poverty in old age [82]. Future studies should seek to fill this research gap for Poland. Furthermore, it appears that having a paid job after retiring plays a different role for different subgroups, especially for men. Thus, a deeper investigation into the relationships between job characteristics, retirement, and subjective quality of life is needed.

## Figures and Tables

**Table 1:** Results of the fixed-effects ordered logit models predicting the happiness levels among people aged 55–69 who retired in Poland.

	Model 1A			Model 1B		
Variables	Total	Men	Women	Total	Men	Women
Age	0.10 ***	0.05	0.14 ***	0.10 ***	0.05	0.14 ***
Disability *(ref. without disability)*						
with disability	0.09	0.07	0.11	0.09	0.06	0.10
Partnership status *(ref. without a spouse/partner)*						
with a spouse/partner	1.17 ***	1.92 **	0.97 *	1.17 ***	1.94 **	0.97 *
Living arrangements *(ref. living alone)*						
living with a spouse only	−0.43	−1.39 ***	−0.13	−0.43	−1.40 ***	−0.13
with a spouse and children	−0.89 **	−1.73 ***	−0.70	−0.89 **	−1.73 ***	−0.70
with children only	−0.39	−1.37	−0.22	−0.39	−1.36	−0.23
with a family of a child, multigenerational households	−0.62	−1.55 ***	−0.37	−0.62	−1.55 ***	−0.37
Satisfaction with the financial situation of one’s family	0.39 ***	0.37 ***	0.40 ***	0.39 ***	0.37 ***	0.40 ***
Satisfaction with one’s health status	0.40 ***	0.35 ***	0.43 ***	0.40 ***	0.35 ***	0.43 ***
Employment status (*ref. not in employment*)						
employed	0.21	−0.02	0.41 *			
Retirement (*ref. no*)						
yes	0.20	0.38	0.09			
Retirement # Employment status (*ref. not retired, nor employed)*						
Retired, not employed				0.20	0.39	0.07
Not retired, in employment				0.21	0.03	0.37
Retired, in employment				0.46	−0.75	1.51
Observations	2606	1095	1511	2606	1095	1511

Also controlled for sex, place of residence, and educational level, which are omitted here due the method employed. Significance level: *** *p* < 0.01, ** *p* < 0.05, * *p* < 0.1. The number of observations differs from the final sample size due to the fact that in this method the estimation sample includes the original sample as well as additional observations (copies of the original data). Source: own estimations based on the data from the Social Diagnosis survey 2007–2015.

**Table 2:** Results of the fixed-effects logit models predicting loneliness among people aged 55–69 who retired in Poland.

	Model A			Model B		
Variables	Total	Men	Women	Total	Men	Women
Age	−0.05	−0.14 **	−0.01	−0.05	−0.14 **	−0.00
Disability *(ref. without disability)*						
with disability	0.08	0.08	0.07	0.09	0.09	0.07
Partnership status *(ref. without a spouse/partner)*						
with a spouse/partner	−1.28 ***	−2.47	−1.07 *	−1.29 ***	−2.48	−1.08
Living arrangements *(ref. living alone)*						
living with a spouse only	−1.23 ***	−0.78	−1.45 **	−1.21 ***	−0.78	−1.42 **
with a spouse and children	−0.79	−0.35	−1.00	−0.76	−0.36	−0.97
with children only	−0.52	2.34	−0.88	−0.50	2.33	−0.86
with a family of a child, multigenerational households	−1.10 **	−1.05	−0.98	−1.07 **	−1.05	−0.95
Satisfaction with one’s family financial situation	−0.24 ***	−0.23 **	−0.25 ***	−0.24 ***	−0.23 *	−0.25 **
Satisfaction with one’s health status	−0.31 ***	−0.44 ***	−0.23 **	−0.31 ***	−0.44 ***	−0.23 ***
Employment status (*ref. not in employment*)						
employed	−0.05	0.19	−0.22			
Retirement (*ref. no*)						
yes	0.07	0.69 **	−0.27			
Retirement # Employment status (*ref. not retired, nor employed)*						
Retired, not employed				0.09	0.72 **	−0.25
Not retired, in employment				0.02	0.24	−0.14
Retired, in employment				−1.48	−12.67 ***	−1.66
*Observations*	1503	567	936	1503	567	936
*Number of respondents*	437	165	272	437	165	272

Also controlled for sex, place of residence, and educational level, which are omitted here due the method employed. Significance level: *** *p* < 0.01, ** *p* < 0.05, * *p* < 0.1. Source: own estimations based on the data from the Social Diagnosis survey 2007–2015.

## Data Availability

Data are available on www.diagnoza.com (accessed 27 July 2021).

## Appendix A

**Table A1:** Descriptive statistics for the control and explanatory variables for the final sample.

Variable	2007	2009	2011	20013	2015
	Mean				
Age	58.70	59.64	61.08	62.50	64.00
Satisfaction with the financial situation of one’s family	3.14	3.28	3.43	3.47	3.69
Satisfaction with one’s health status	3.13	3.27	3.44	3.45	3.45
	Proportion				
Sex					
Men	46.5	42.3	44.3	43.1	39.2
Women	53.5	57.7	55.7	56.9	60.8
Place of residence					
Urban	50.0	51.7	52.1	51.8	51.1
Rural	50.0	48.4	47.9	48.2	48.9
Disability					
Without disability	64.8	69.1	72.0	72.5	78.7
With disability	35.2	30.9	28.0	27.6	21.3
Partnership status					
Without a spouse/partner	20.0	24.6	24.6	23.9	29.3
With a spouse/partner	80.0	75.4	75.4	76.1	70.7
Living arrangements					
With a spouse only	32.6	37.7	37.6	42.2	43.6
With a spouse and children	36.1	29.2	27.2	20.8	17.9
With children only	11.3	9.0	7.2	9.9	10.2
With a family of a child, multigenerational households	14.8	13.8	15.8	17.9	12.6
Living alone	5.2	10.3	12.2	9.3	15.7
Level of education					
Primary and lower	32.6	32.7	31.4	29.9	26.9
Basic vocational and junior secondary	30.4	32.2	32.5	34.5	36.8
Secondary	23.0	23.4	23.4	22.6	22.8
Higher and post-secondary	13.9	11.8	12.7	13.0	13.6
Employment status					
Not employed (unemployed or inactive)	58.3	66.7	74.1	86.0	98.6
In employment	41.7	33.3	26.0	14.1	1.5
Retirement					
No	58.3	66.7	74.1	86.0	98.6
Yes	41.7	33.3	26.0	14.1	1.5
Happiness					
Very unhappy	5.7	6.8	4.0	2.0	2.4
Rather unhappy	44.4	43.6	30.1	31.0	27.1
Rather happy	45.7	41.4	59.6	55.5	60.5
Very happy	4.4	8.3	6.4	11.5	9.9
Loneliness					
No	75.4	71.7	72.8	77.1	75.8
Yes	24.6	28.3	27.2	22.9	24.2
Observations	141	336	391	355	280

Source: Own estimation based on data from the Social Diagnosis survey 2007–2015.
